# Insulin Regulates Glucose Consumption and Lactate Production through Reactive Oxygen Species and Pyruvate Kinase M2

**DOI:** 10.1155/2014/504953

**Published:** 2014-05-08

**Authors:** Qi Li, Xue Liu, Yu Yin, Ji-Tai Zheng, Cheng-Fei Jiang, Jing Wang, Hua Shen, Chong-Yong Li, Min Wang, Ling-Zhi Liu, Bing-Hua Jiang

**Affiliations:** ^1^Department of Pathology, State Key Lab of Reproductive Medicine, Cancer Center, Nanjing Medical University, Nanjing 210029, China; ^2^Department of Pathology, Anhui Medical University, Hefei 230032, China; ^3^Department of Oncology, The First Affiliated Hospital of Nanjing Medical University, Nanjing 210029, China; ^4^Department of Pathology, Anatomy and Cell Biology, Thomas Jefferson University, Philadelphia, PA 19107, USA

## Abstract

Although insulin is known to regulate glucose metabolism and closely associate with liver cancer, the molecular mechanisms still remain to be elucidated. In this study, we attempt to understand the mechanism of insulin in promotion of liver cancer metabolism. We found that insulin increased pyruvate kinase M2 (PKM2) expression through reactive oxygen species (ROS) for regulating glucose consumption and lactate production, key process of glycolysis in hepatocellular carcinoma HepG2 and Bel7402 cells. Interestingly, insulin-induced ROS was found responsible for the suppression of miR-145 and miR-128, and forced expression of either miR-145 or miR-128 was sufficient to abolish insulin-induced PKM2 expression. Furthermore, the knockdown of PKM2 expression also inhibited cancer cell growth and insulin-induced glucose consumption and lactate production, suggesting that PKM2 is a functional downstream effecter of insulin. Taken together, this study would provide a new insight into the mechanism of insulin-induced glycolysis.

## 1. Introduction

Insulin is known to play an important role in human glucose metabolism [1]. Many human diseases cause glucose metabolism disorders such as diabetes and cancer [2, 3]. However, the molecular mechanisms of insulin in regulating glucose metabolism of cancer remain to be elucidated. Changes of rate-limiting glycolytic enzymes are observed during cancer metabolism. Among these enzymes, pyruvate kinase (PK) plays a crucial role in catalyzing the formation of pyruvate and ATP from phosphoenolpyruvate and ADP [4, 5]. There are four isoforms of PK in mammals, PKL, PKR, PKM1, and PKM2. PKL, PKR, and PKM1 are tissue-specific isoenzymes, whereas PKM2 is considered an embryonic and cancer cell-specific isoform [6]. Evidence supports that the loss of the tissue-specific isoenzymes and subsequent expression of PKM2 are involved in tumor initiation as well as malignant progression. Knockdown of PKM2 expression or the replacement of PKM2 with PKM1 has been demonstrated to inhibit cancer metabolism and tumor growth [5, 7]. Transcription factors such as HIF-1*α* and other genes relevant to tumorigenesis are potent PKM2 activators, while a number of genes associated with cell proliferation, metabolism, and tumor growth are downstream targets of PKM2 [8–10].

Lines of evidence in recent years have suggested a crucial role of reactive oxygen species (ROS) in cancer cellular functions [11]. High levels of endogenous ROS production are associated with cancer development [12, 13]. ROS, especially hydrogen peroxide (H_2_O_2_), are also induced by a variety of external stimulators including growth factors such as insulin [14]. However, the role of ROS production in cancer cells in response to insulin-induced glucose metabolism remains to be elucidated.

Recently, miRNAs are known to be involved in many human diseases, such as diabetes and cancer [15, 16]. miRNAs are small, noncoding RNAs that have been confirmed to be a new kind of gene expression regulators through negatively regulating protein-coding genes. The causal roles of miRNAs in cancer have been well documented and miRNA-based anticancer therapies are in development [17, 18]. Several miRNAs with evident roles in cancer are reported to participate in insulin and ROS signaling pathways. For example, Let-7 family regulates multiple aspects glucose metabolism in multiple organs [19]; miR-143 regulates glucose metabolism of cancer cells by targeting hexokinase 2 isoform (HK2) [20]; miR-21 is an important target of ROS [21]. Despite these studies, whether or not miRNAs take part in insulin-induced PKM2 expression and the underlying mechanisms by which PKM2 exerts effects in this pathology remain unclear.

In the present study, we plan to study whether (1) ROS are involved in insulin-regulated glycolysis in hepatocellular carcinoma cells; (2) insulin regulates PKM2 expression via ROS production; (3) insulin upregulates PKM2 expression in ROS dependent manner through miRNAs expression; and (4) PKM2 is required for insulin-induced aerobic glycolysis. These studies will determine the role of ROS/miRNAs/PKM2 in mediating insulin effects and are helpful to understand the mechanism of insulin in regulating hepatocellular carcinoma cell glycolysis.

## 2. Materials and Methods

### 2.1. Reagents and Cell Culture

Human HepG2 hepatocellular carcinoma cells were obtained from American Type Culture Collection (Manassas, VA, USA). Human hepatocellular carcinoma cell lines BeL7402 were obtained from the Cell Bank of the Chinese Academy of Science (Shanghai, China). Cells were cultured in Dulbecco's modified Eagle's medium (DMEM) supplemented with 5% FBS, penicillin (100 U/mL), and streptomycin (100 *μ*g/mL) at 37°C in 5% CO_2_ incubator. Trypsin (0.25%)/EDTA solution was used to detach the cells for subculturing the cells. Insulin was purchased from Sigma (St. Louis, MO).

### 2.2. ROS Measurement

HepG2 cells were seeded in a 12-well plate at 8 × 10^4^ cells/well and cultured at 37°C for 24 h. The cells were then incubated in serum-free medium for 24 h, followed by the pretreatment with catalase (1500 U/mL) for 1 h. CM-H2DCFDA (5 *μ*M) (Invitrogen, USA) was added and incubated with the cells for 10 min. Cells were then stimulated with insulin (200 nM) for 10 min. The cells were washed twice with phosphate-buffered saline (PBS) and fixed with 10% buffered formalin. The images were captured with a fluorescence microscope.

### 2.3. Malondialdehyde (MDA) Analysis

MDA levels in the cells were determined by the thiobarbituric acid (TBA) method using an assay kit according to manufactory guidance (Beyotime Biotechnology, Shanghai, China). Briefly, protein samples were incubated with TBA at 100°C for 15 min, followed by a centrifugation at 1000 ×g for 10 min. Supernatants were transferred to a 96-well plate, and the absorbance was measured at 532 nm. The MDA levels were analyzed and normalized to each sample protein concentration.

### 2.4. Measurement of Glucose Consumption and Lactate Production

A total of 5 × 10^4^ cells per well were seeded in 24-well plates and treated as above. Cells were trypsinized and counted, while the supernatants of cell culture medium were collected. The media were assayed immediately for glucose and lactate levels by using glucose assay kit and lactate assay kit (Biovision, Mountain View, CA) according to the manufacturer's instruction. The glucose consumption and lactate production were normalized to cell number. The experiments were performed with three replicates and repeated for three times.

### 2.5. Real-Time RT-PCR

Total RNAs from cells were isolated using TRIzol (Invitrogen, USA) according to the manufacturer's instruction. cDNA synthesis was performed with 1 *μ*g total RNAs using PrimeScriptTM RT reagent kit (Takara, China). Aliquots of these cDNAs were used for quantitative real-time PCR using SYBR Premix DimerEraser (Takara, China). Expression levels of miR-145 and miR-128 were normalized to U6 levels, expression levels of PKM2 were normalized to GAPDH level for each sample, and fold changes were calculated by relative quantification (2^−ΔΔCt^). Primers used were listed in Supplementary Table 1 in Supplementary Material available online at http://dx.doi.org/10.1155/2014/504953.

### 2.6. Western Blotting and Antibodies

Cells were harvested and lysed in radioimmunoprecipitation assay (RIPA) buffer supplemented with proteinase inhibitors cocktail. The protein extracts were separated by SDS-polyacrylamide gel electrophoresis (SDS-PAGE), transferred to nitrocellulose membranes, and incubated with antibodies against PKM2 (Signalway Biotechnology, Pearland, TX), HIF-1*α* (BD Biosciences, Sparks, MD), p70S6K1 (Cell Signaling Technology, Danvers, MA), and GAPDH (Sigma, St. Louis, MO). The protein bands were detected by incubating with horseradish peroxidase- (HRP-) conjugated antibodies and visualized using the Super Signal West Pico Chemiluminescent Substrate Kits (Thermo Scientific, Rockford, IL).

### 2.7. Transient Transfection

Double strands miR-145 and miR-128 and scrambled control precursors were synthesized by Gene-pharma (Shanghai, China). HepG2 and Bel7402 cells were transfected with miR-145, miR-128, or scramble control precursor by Lipofectamine 2000 (Invitrogen, USA) according to the manufacturer's instruction. The sequences of miRNA precursors were listed in Supplementary Table 2.

Small interfering RNA (siRNA) duplex oligonucleotides targeting human PKM2 (siPKM2) or scrambled control (siSCR) were purchased from GenePharma (Shanghai, China). HepG2 and Bel7402 cells were transfected with siPKM2 or siSCR using Lipofectamine RNAiMax (Invitrogen) in serum-free Opti-MEM according to the manufacturer's instruction. The sequences of small interfering RNA for PKM2 were listed in Supplementary Table 3.

### 2.8. Cell Proliferation Assay

HepG2 and Bel7402 cells were transfected with siPKM2 or the scrambled control and cultured at 37°C for 24 h. The cells were then trypsinized, resuspended, and seeded in a 96-well plate at 3000 cells per well. The cell proliferation was measured at 12 h, 24 h, 48 h, 72 h, and 96 h using a Cell Counting Kit-8 (CCK-8) (Dojindo Laboratories, Kumamoto, Japan) according to the manufacturer's instruction. All experiments were performed in triplicate and were repeated for three times.

### 2.9. Statistical Analysis

Numerical results were presented as mean ± SD. Statistical analysis was performed based on a Student's *t*-test at the significance level of *P* < 0.05 using GraphPad Prisms Software.

## 3. Results 

### 3.1. Evidence for the Involvement of ROS in Insulin-Regulated Glycolysis in Hepatocellular Carcinoma Cells

**Figure 1:**
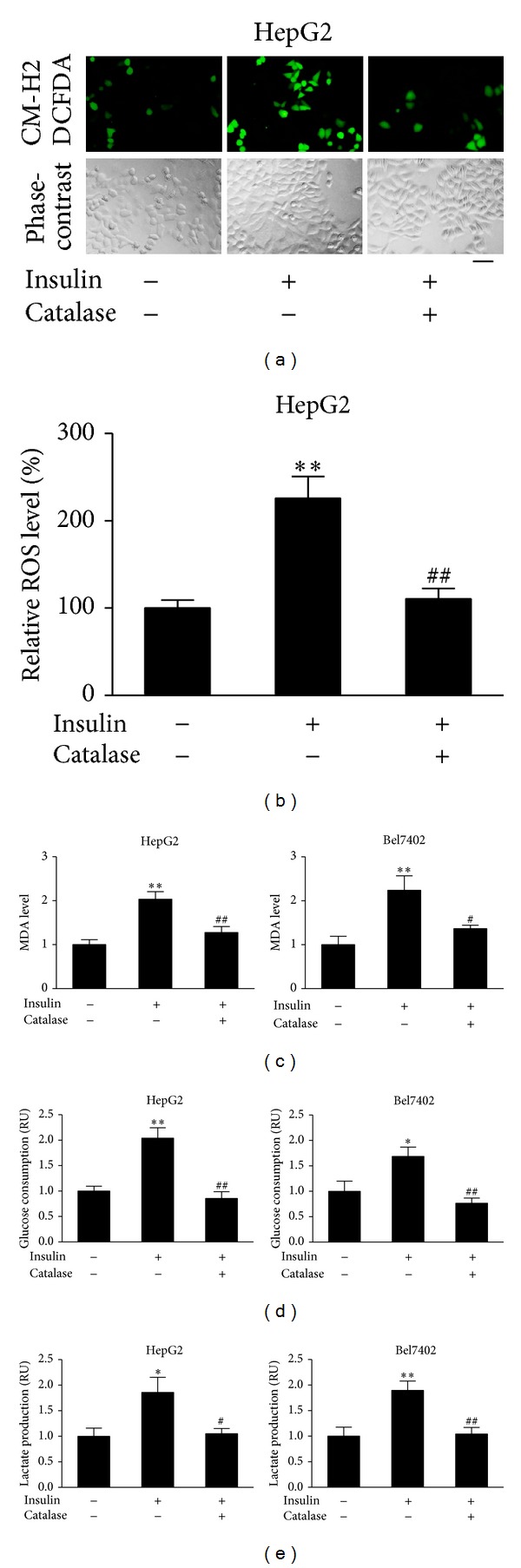
Insulin promoted MDA production, glucose consumption and lactate production through ROS production in hepatocellular carcinoma cells. (a) HepG2 cells were seeded in a 12-well plate at 8 × 10^4^ cells/well and cultured at 37°C for 24 h. The cells were then incubated in serum-free medium for 24 h, followed by the pretreatment with catalase (1500 U/mL) for 1 h. CM-H2DCFDA (5 *μ*M) was added and incubated with the cells for 10 min. Cells were then stimulated with insulin (200 nM) for 10 min. The cells were washed thrice with 1x PBS. The representative images were captured with a fluorescence microscope. Bar, 50 *μ*m. (b) Levels of ROS fluorescence signals were quantified by ImageJ; **P < 0.01 compared with that of the same cell line treated without insulin and catalase; ^##^ *P* < 0.01 compared to the cells treated with insulin alone. (c) HepG2 and Bel7402 cells were cultured in serum-free medium overnight. Then, the cells were treated with catalase (1500 U/mL) for 1 h, followed by insulin treatment (200 nM) for 12 h. The proteins were collected and subjected to MDA analysis. Data were presented by mean ± SD (*n* = 3). ** Significant difference compared with that of the same cell line treated or without insulin and catalase (*P* < 0.01); ^#^ and ^##^ significant difference compared to the cells treated with insulin alone (*P* < 0.05 and *P* < 0.01, resp.). (d) and (e) HepG2 cells and Bel7402 cells were seeded in 24-well plates and cultured in serum-free medium for 24 h, followed by the treatment with catalase (1500 U/mL) for 1 h. Cells were then stimulated with insulin (200 nM) for 12 h. The medium was collected, and the glucose consumption and lactate production levels were analyzed. Data were mean ± SD from three independent experiments. **P < 0.01 and *P < 0.05 compared with that of the same cell line treated without insulin and catalase;^ #^ *P* < 0.05 and ^##^ *P* < 0.01 compared to the cells treated with insulin alone.

Although insulin has been associated with a variety of cancers and liver cell functions [22, 23], and ROS as secondary messengers mediate the cancers signals, it remains unknown whether ROS participate in transmitting insulin signaling. To this end, ROS levels in response to insulin treatment were analyzed in HepG2 cells. The cells were cultured in serum-free medium, followed by the treatment of insulin. As shown in Figures 1(a) and 1(b), treatment of cells with insulin led to higher ROS levels. Addition of ROS scavenger catalase blocked the effect of insulin treatment. Further experiments were performed in HepG2 and Bel7402 cells using MDA analysis, a stable indicator of oxidative stress. The results showed that MDA levels in the insulin treatment were increased approximately 2-fold when compared with that in the cells without insulin treatment. Catalase administration significantly decreased insulin-induced MDA level (Figure 1(c)). These data suggest that insulin is able to induce ROS production.

Next, we examined the effect of ROS on insulin-regulating glucose energy metabolism. Insulin dramatically increased glucose consumption and lactate production in HepG2 and Bel7402 cells. Administration of catalase attenuated the increase in glucose metabolism induced by insulin (Figures 1(d) and 1(e)). Thus, it appears that ROS participate in insulin-regulated glycolysis.

### 3.2. Insulin Induced PKM2 Expression through ROS Production

**Figure 2:**
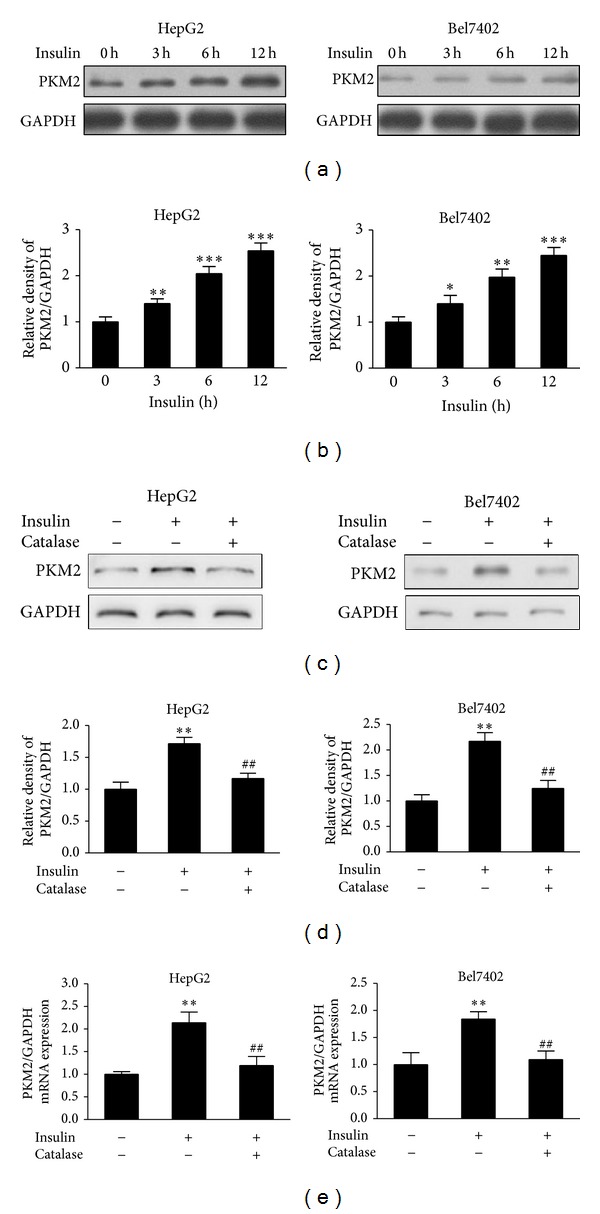
Insulin-induced PKM2 expression was inhibited by catalase. (a) HepG2 cells and Bel7402 cells were cultured to 70% confluence and then starved in serum-free medium for 24 h. The cells were exposed to 200 nM of insulin for 0 h, 3 h, 6 h, and 12 h. PKM2 levels were determined by immunoblotting. (b) Relative densities of PKM2/GAPDH from three independent experiments were normalized to those of control and presented as mean ± SD. * Significant difference compared to control without insulin treatment (*P* < 0.05). (c) The starved HepG2 cells and Bel7402 cells were pretreated with catalase (1500 U/mL) for 1 h, followed by stimulation with insulin (200 nM) for 6 h. Protein expression was determined by immunoblotting. (d) Results were expressed as a percentage of the control cultures and were the mean ± SD from three replications. (e) HepG2 cells and Bel7402 cells were treated as in (c). Total RNAs were extracted and used for real-time RT-PCR for detecting PKM2 and GAPDH mRNA levels. ** Significant difference compared to the control without insulin and catalase treatment treated without insulin and catalase treatment (*P* < 0.01); ^##^ significant difference compared to the cells treated with insulin alone (*P* < 0.01).

Pyruvate kinase M2 (PKM2), the key kinase, catalyzes the last reaction of glycolysis and converts phosphoenolpyruvate (PEP) to pyruvate producing ATP. The expression levels of PKM2 are used as one of the important metabolic signatures of tumor cells. To test whether insulin promotes glycolysis through regulating PKM2 expression, HepG2 cells and Bel7402 cells were cultured in serum-free medium for 24 h and exposed to 200 nM of insulin for 0 h, 3 h, 6 h, and 12 h. The immunoblotting results showed that insulin significantly induced PKM2 expression in a time-dependent manner (Figures 2(a) and 2(b)).

Since we showed that ROS were induced by insulin, we tested whether insulin induced PKM2 expression through ROS production. Pretreatment of HepG2 and Bel7402 cells with catalase greatly suppressed the PKM2 protein levels induced by insulin (Figures 2(c) and 2(d)). Further experiments were performed by using real-time PCR to analyze PKM2 mRNA expression levels. Similarly, PKM2 mRNA levels were enhanced in response to insulin treatment and decreased by catalase treatment (Figure 2(e)). This result suggests that insulin-induced PKM2 expression requires ROS production.

### 3.3. Insulin Upregulates PKM2 Expression in ROS Dependent Manner through miR-145 and miR-128 Expression

**Figure 3:**
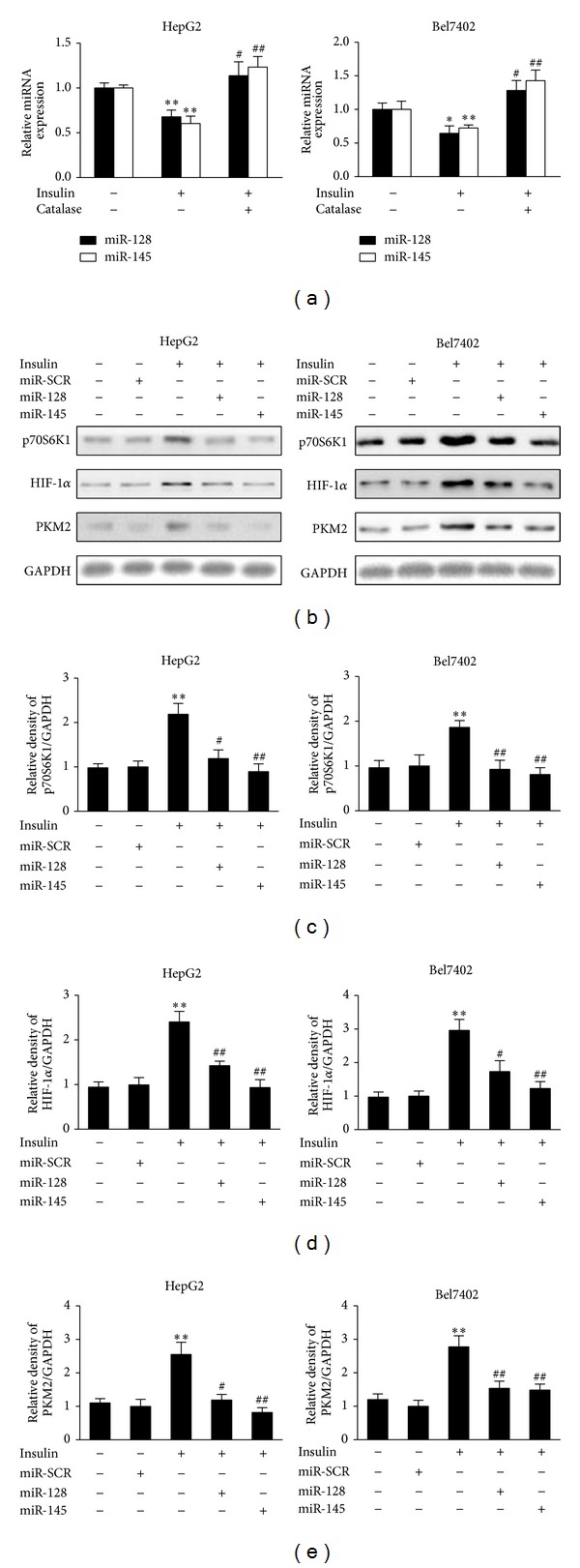
miR-145 and miR-128 are suppressed by insulin and involved in insulin-induced PKM2 expression. (a) HepG2 cells and Bel7402 cells were cultured overnight and switched to serum-free medium for 20 h. The starved cells were pretreated with catalase (1500 U/mL) for 1 h. Insulin (200 nM) was added and the cells were incubated for 6 h. Total RNAs were extracted and used for real-time RT-PCR for detecting the expression levels of miR-145, miR-128, and U6. * and ** Significant difference compared to control (*P* < 0.05 and *P* < 0.01); ^##^ significant difference compared to treatment with insulin alone (*P* < 0.01). (b) HepG2 cells and Bel7402 cells were transfected with miR-145, miR-128, or miRNA scrambled control precursor. After transfection for 24 h, cells were cultured in serum-free medium for 20 h and treated without or with insulin (200 nM) for 6 h. Protein expression levels of p70S6K1, HIF-1*α*, PKM2, and GAPDH were determined by immunoblotting. (c), (d), and (e) Relative protein densities were quantified using ImageJ software. Results are presented as mean ± SD from three independent experiments. * and ** Significant difference compared to the value of the scramble control (*P* < 0.05 and *P* < 0.01); ^#^ and ^##^ significant difference compared to that treated with insulin alone (*P* < 0.05 and *P* < 0.01).

There is accumulating evidence for the miRNAs expression which may be altered in response to exogenous agents that, at least in part, induce intracellular insulin and oxidative stress [21, 24]. Hydrogen peroxide treatment suppressed miR-145 and miR-128 expression (Figure S1), suggesting that ROS inhibit miR-145 and miR-128 expression. To test whether insulin affect miR-145 and miR-128 expression through ROS, we showed that pretreatment of HepG2 and Bel7402 cells with catalase greatly induced the expression levels of miR-145 and miR-128 suppressed by insulin (Figure 3(a)). This result suggests that insulin-regulated expression of miR-145 and miR-128 requires ROS production.

Our previous studies demonstrated that p70S6K1 is a direct target of miR-145 and miR-128 in ovarian cancer cells and glioma cells [25, 26]. We detected p70S6 K1 and HIF-1*α* levels in response to insulin treatment. As shown in Figures 3(b), 3(c), and 3(d), p70S6K1 and HIF-1*α* protein levels were increased after insulin treatment, and addition of miR-128 or miR-145 precursors inhibited insulin-induced p70S6K1 and HIF-1*α* expression. This result indicates that miR-128 or miR-145 is required for insulin-induced expression of p70S6K1 and HIF-1*α*. Since we showed that miR-128 and miR-145 are required for insulin-induced p70S6K1 and HIF-1*α* expression, we tested whether insulin-suppressing miR-128 and miR-145 could play a role in PKM2 expression in HepG2 and Bel7402 cells. To test this, the cells were pretreated with miR-128 and miR-145 and then stimulated with insulin. miR-128 and miR-145 treatment inhibited insulin-induced expression of PKM2 (Figures 3(b) and 3(e)), indicated that miR-128 and miR-145 are required for insulin-induced PKM2 expression.

### 3.4. PKM2 Is Critical for Insulin-Induced Aerobic Glycolysis and Cell Growth

**Figure 4:**
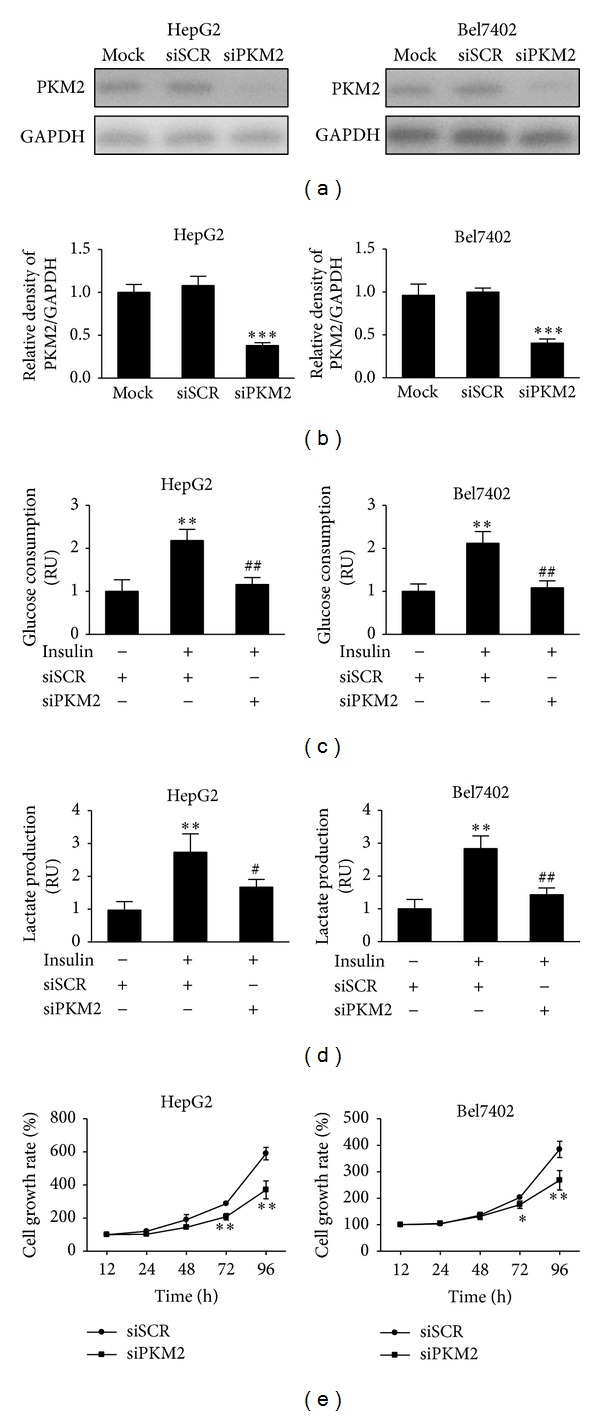
Knockdown of PKM2 is sufficient to inhibit insulin-induced glucose consumption, lactate production, and cell proliferation. (a) HepG2 and Bel7402 cells were transfected with siRNA against PKM2 (siPKM2) or scramble control (siSCR) for 48 h. The protein levels of PKM2 were analyzed by immunoblotting. (b) Relative densities of PKM2/GAPDH from three independent experiments were normalized to those of control and presented as mean ± SD. *** indicate significant difference when compared to scramble control at *P* < 0.001. (c) and (d) HepG2 and Bel7402 cells were transfected with siRNA against PKM2 (siPKM2) or scramble control (siSCR) for 24 h, followed by starving in serum-free medium for 24 h. Then, cells were stimulated with insulin (200 nM) for 12 h. Cells were trypsinized and counted, while the medium was collected. The glucose consumption and lactate production levels were analyzed. Data were mean ± SD from three independent experiments. **P < 0.01 significant difference when compared to cell treated with siSCR; ^#^ *P* < 0.05 and ^##^ *P* < 0.01 significant difference compared to cell treated with siSCR and insulin. (e) HepG2 and Bel7402 cells were transfected with siRNA against PKM2 (siPKM2) or scramble control (siSCR) for 24 h. The cells were then trypsinized and resuspended. Cells at 3000 cells per well in a 96-well plate. The cell proliferation was measured at 12 h, 24 h, 48 h, 72 h, and 96 h. Values represent means ± SD. * and ** Compared to scramble control (*P* < 0.05 and *P* < 0.01).

To test whether the inhibition of PKM2 expression attenuates insulin-induced glycolysis, HepG2 and Bel7402 cells were transfected with siRNA against PKM2 or scrambled control siRNA. After transfection with siPKM2, the expression levels of PKM2 were markedly inhibited by 70–80% when compared to scrambled control (Figures 4(a) and 4(b)). Insulin increased glucose consumption and lactate production by 2-fold when compared to control group without insulin treatment, while PKM2 knockdown inhibited insulin-induced glucose consumption and lactate production to 50% and 60%, respectively (Figures 4(c) and 4(d)). These results confirm that PKM2 is an important regulator in insulin-regulating glycolysis.

Given that the energy metabolism is critical to the survival and proliferation of cancer cells, we examined the effect of PKM2 on cell proliferation. When compared to siSCR treatment, inhibition of PKM2 in HepG2 and Bel7402 cells inhibited cell proliferation after the culture for 3-4 days, suggesting that PKM2 affects cell growth* in vitro* (Figure 4(e)). Taken together, these data indicate that PKM2 is critical for cell growth.

## 4. Discussion

Insulin has been shown to induce glucose metabolism and associated with a variety of cancer development in solid tumors [1, 27]. However, the mechanisms of insulin in glucose metabolism in cancer cells have not been directly examined. PKM2 is the last rate-limiting glycolytic enzymes of the glycolytic metabolism, which is preferentially expressed in embryonic tissue and cancer cells [4, 5]. Studies demonstrate that during tumor initiation as well as malignant progression PKM1 disappear and PKM2 reappears which leads to the switch from regular cell metabolism to aerobic glycolysis [28, 29]. Previous researches have shown that PKM2 may be induced by transcription factors, such as HIF-1*α*, while a number of genes associated with cell proliferation, metabolism, and tumor growth are downstream targets of PKM2.

Recent evidence has demonstrated the importance of ROS as secondary messengers in a variety of cellular functions [11–13]. Here, we present evidence that ROS promoted the effects of insulin-induced glycolysis and PKM2 expression in the cultured hepatocellular carcinoma cells. In general, insulin led to an increase in ROS levels and addition of catalase blocked the effect of insulin treatment. This result is consistent with our previous study of insulin-induced generation of H_2_O_2_ in PC-3 cells [14]. More importantly, ROS levels were positively correlated with insulin-induced glycolysis. PKM2 has been reported essential for glycolytic metabolism and insulin stimulates expression of the PKM2 [5, 28]. We further explored the role of ROS in the insulin-induced PKM2 expression. Results showed that insulin significantly induced PKM2 protein and mRNA expression levels and catalase greatly suppressed the effect induced by insulin. Expression levels of PKM2 protein and mRNA were affected, indicating the possible involvement of other factors in insulin-induced PKM2 expression.

Several miRNAs are reported to be altered in response to exogenous agents such as insulin and ROS [19–21]. In this study, we demonstrated that insulin suppressed expression levels of miR-145 and miR-128 through ROS production. Our previous studies showed that miR-145 and miR-128 inhibit HIF-1*α* expression by directly targeting p70S6K1 [25, 26]. Human* PKM2* gene sequence revealed a candidate HRE within the first intron containing the HIF-1 binding site 5′-ACGTG-3′ followed by a 5′-CACA-3′ sequence, which is found in many HREs [9, 30]. Correlation between miR-145 and miR-128 expression and insulin-regulated PKM2 expression further support our conclusion that insulin upregulates PKM2 expression through miR-145 and miR-128 expression. However, the mechanism by which insulin and ROS inhibit miR-145 and miR-128 is currently unknown. One possibility is that insulin and ROS may affect miR-145 and miR-128 expression through DNA hypermethylation. DNA hypermethylation has been shown to be associated with aberrant miRNA expression profiles in cancer [31–33]. Further studies are needed to address how insulin and ROS activate miR-145 and miR-128 in HepG2 and Bel7402 cells.

In addition, we have shown that knockdown of PKM2 expression decreased insulin-induced aerobic glycolysis and cancer cell proliferation. This possibly explains the link between high insulin levels and elevated cancer risk. Moreover, this study may provide some useful information that PKM2 may act as a potential strategy for therapeutic purpose in liver cancer treatment in the future.

In conclusion, the results from the present study indicate that ROS promoted the effects of insulin-induced glycolysis and PKM2 expression in human hepatocellular carcinoma cells, that insulin upregulates PKM2 expression in ROS dependent manner through miR-145 and miR-128 suppression, and that PKM2 is important for insulin-induced aerobic glycolysis and cell proliferation. Our results contribute to understanding the role of insulin in cancer metabolism and also providing new insights into the role of PKM2 in pathogenesis of liver cancer.

## Supplementary Material

MiR-128 and MiR-145 Are Inhibited by ROS. There is accumulating evidence for the miRNA expression which may be altered in response to exogenous agents that, at least in part, induce intracellular insulin and oxidative stress [21, 24]. We treated hepatocellular carcinoma cells using hydrogen peroxide, and found that miR-128 and miR-145 expression levels were inhibited by hydrogen peroxide in both HepG2 and Bel7402 cells (Figure S1). This result suggests that ROS inhibit miR-128 and miR-145 expression.
Supplementary figure legend: FIGURE.S1. HepG2 cells and Bel7402 cells were cultured in serum-free medium overnight. The cells were treated with 50 µM H2O2 for 6 h. Total RNAs were extracted and used for real time RT–PCR analysis for detecting the expression levels of miR-145, miR-128 and U6. ∗∗ indicates significant difference compared to the control (p< 0.01).
