# Supplementary material for: Insulin Regulates Glucose Consumption and Lactate Production through Reactive Oxygen Species and Pyruvate Kinase M2
Source: Oxid Med Cell Longev. 2014 May 8;2014:504953. doi: 10.1155/2014/504953 (PMC4034658; doi:10.1155/2014/504953)
Supplement: Supplementary file 1 — MiR-128 and MiR-145 Are Inhibited by ROS. There is accumulating evidence for the miRNA expression which may be altered in response to exogenous agents that, at least in part, induce intracellular insulin and oxidative stress [21, 24]. We treated hepatocellular carcinoma cells using hydrogen peroxide, and found that miR-128 and miR-145 expression levels were inhibited by hydrogen peroxide in both HepG2 and Bel7402 cells (Figure S1). This result suggests that ROS inhibit miR-128 and miR-145 expression. Supplementary figure legend: FIGURE.S1. HepG2 cells and Bel7402 cells were cultured in serum-free medium overnight. The cells were treated with 50 µM H2O2 for 6 h. Total RNAs were extracted and used for real time RT–PCR analysis for detecting the expression levels of miR-145, miR-128 and U6. ∗∗ indicates significant difference compared to the control (p< 0.01). [file 504953.f1.zip › mat.504953.v1.pdf]

Table 1. List of primers used in real-time RT-PCR experiments

miR-145-stem-loop RT primer	5'- CTCAACTGGTGTCGTGGAGTCGGCAATTCAGTTGAGAAGGGATT -3'
miR-145-forward	5'- ACACTCCAGCTGGGGTCCAGTTTTCCCAGGAA -3'
miR-145-reverse	5'- GTTCGCTGAGATGAAGCACTG -3'
miR-128-stem-loop RT primer	5'- CTCAACTGGTGTCGTGGAGTCGGCAATTCAGTTGAGAAAAGAGA -3'
miR-128-forward	5'- ACACTCCAGCTGGGTCACAGTGAACCGGTC -3'
miR-128-stem-loop RT primer	5'- TGGTGTCGTGGAGTCG -3'
U6-stem-loop RT primer	5'- AACGCTTCACGAATTTGCGT -3'
U6-forward	5'- CTCGCTTCGGCAGCACA -3'
U6-reverse	5'- TGGTGTCGTGGAGTCG -3'
PKM2-forward	5'- CGAGCCTCAAGTCACTCCAC -3'
PKM2-reverse	5'- GTGAGCAGACCTGCCAGACT-3'
GAPDH-forward	5'-ATGGGTGTGAACCATGAGAAGTATG-3'
GAPDH-reverse	5'-GGTGCAGGAGGCATTGCT-3'

Supplementary Table 1

Table 2. List of the precusor sequences

miR-145 mimics (sense)	5'- GUCCAGUUUUCCCAGGAAUCCCU -3'
miR-145 mimics (antisense)	5'- UUCAGGUCAAAAGGGUCCUUAGG -3'
miR-128 mimics (sense)	5'- UCACAGUGAACCGGUCUCUUU -3'
miR-128 mimics (antisense)	5'- AGAGACCGGUUCACUGUGAUU -3'
scrambled precursor (sense)	5'- UUCUCCGAACGUGUCACGUTT -3'
scrambled precursor (antisense)	5'- ACGUGACACGUUCGGAGAATT -3'

Supplementary Table 2

Table 3. List of the Small interfering RNA sequences	
siPKM2 (sense)	5'- GCTGTGGCTCTAGACACTATT -3'
siPKM2 (antisense)	5'- CGACACCGAGATCTGTGATTT-3'

Supplementary Table 3
